# How Abusive Supervision Affects Employees’ Unethical Behaviors: A Moderated Mediation Examination of Turnover Intentions and Caring Climate

**DOI:** 10.3390/ijerph16214187

**Published:** 2019-10-29

**Authors:** Shubo Liu, Qianlin ZHU, Feng Wei

**Affiliations:** 1 Business School, Central University of Finance and Economics, Beijing 100081, China; 2 School of Economics and Management, Tongji University, Shanghai 200092, China; zhuqianlin@tongji.edu.cn

**Keywords:** abusive supervision, turnover intentions, unethical behavior, caring climate

## Abstract

Drawing on psychological contract theory, this research contributes to the unethical behavior literature by exploring employees’ turnover intentions as a mediator of the relationship between abusive supervision and employees’ unethical behavior and the moderating role of the caring climate in the relationship between turnover intentions and unethical behavior. The results from a sample of 679 reveal that turnover intentions mediate relationship between abusive supervision and subordinates’ unethical behavior, and caring climate moderates the positive relationship between turnover intentions and subordinates’ unethical behavior. We also find that the indirect effect is moderated by the caring climate. The theoretical and practical implications are discussed.

## 1. Introduction

Over the years, several attempts have been made in the behavioral ethics literature to explain unethical decision making and behaviors [1,2,3,4]. In accordance with this trend, the vast majority of research has focused on identifying the precursors of unethical behaviors. Among these scholarly efforts, there are three main perspectives: The Bad Apple Perspective, the Bad Barrel Perspective, and the Interactionist Perspective [2,3]. The “bad apples” argument attributes unethical behavior in the organization to a few unsavory individuals [5] who lack some personal quality, such as moral character. This approach prioritizes individual differences and treats measures such as individuals’ economic value orientation, political value orientation, Machiavellianism, and cognitive moral development as significantly related to ethical decision-making behavior [6,7,8]. In comparison, the “bad barrel” argument assumes that something in the organizational environment poisons otherwise good “apples” and attributes unethical behavior to competition, management’s results orientation, a lack of reinforcement of ethical behavior, requests from authority figures to behave unethically, peer behavior, etc. [3]. The interactionist model of unethical behavior in organizations uses a multiple-influences perspective. According to the interactionist perspective, the individual reacts to an ethical dilemma, and the individual’s cognitive moral development stage determines how an individual think about the dilemma [2]. This model assumes that it is not enough to explain or predict ethical decision-making behavior based only on individuals’ cognition of right and wrong or contextual determinants. Instead, additional individual and situational variables interact with the cognitive component to determine how an individual is likely to behave in response to an ethical dilemma.

Informed by the above theoretical models, recent studies have begun to focus on how the role of supervisors or leaders affects subordinates’ unethical behavior, such as theft and other unlawful conduct at the workplace, failure to honor commitments, deliberate deception, and violation of conscience [9,10,11]. However, these studies focus mainly on the role of ethical leadership in inhibiting the unethical behavior of subordinates. That is, existing knowledge on the role of leadership in employee unethical behavior remains incomplete because little is known as to whether the dark side of leadership in general may affect unethical behavior of employees. Thus, further studies are needed to find the triggering or activating effects of negative leadership on employees’ unethical behavior. 

Abusive supervision is one such trigger or activator. It was defined by Tepper [12] as “subordinates’ perceptions of the extent to which supervisors engage in the sustained display of hostile verbal and nonverbal behaviors, excluding physical contact.” Given the prevalence and far-reaching impact of abusive supervision [13], illuminating the influence of abusive supervision on subordinates’ unethical behavior is of theoretical and empirical significance. However, to our knowledge, only a small number of studies have focused directly on the associations between abusive supervision and subordinate unethical behavior. For example, research that draw from social cognitive theory demonstrates that abusive supervision can be associated with subordinates’ unethical behavior [14], and based on trait activation theory, Greenbaum et al. [8] verified the person—(i.e., Machiavellianism) situation (i.e., abusive supervision) interactionist model to predict unethical behavior in organizations. Though these studies have made some contributions to the abusive and unethical behavior literatures, the processes underlying this perception-to-behavior link and the boundary conditions of these processes remain unclear. This is an unfortunate oversight both practically and theoretically. Practically, individual unethical behavior hinders the development of organization [15], and leader behaviors also play an important role in the growth and prohibition of unethical behavior. Theoretically, in view of extant abusive supervision and unethical behavior research, we expect abusive supervision to increase subordinates’ unethical behavior. 

In order to explain the processes underlying abusive supervision and unethical behavior, we draw from psychological contract theory [16] to theorize that subordinates’ turnover intentions mediate the indirect relationship between abusive supervision and unethical behavior. According to psychological contract theory [16], psychological contract refers to “employees’ perceptions of what they owe to their employers and what their employers owe to them” [17]. Employees with intentions to leave are likely to psychologically detach themselves from the organization [18], and the weak psychological contract results in negative employee outcomes such as unethical behavior [19]. Researchers have also argued that abused employees are more inclined to show turnover intentions [20] and that these employees are unlikely to feel obligated to stay in the organization. Such detachments may drive employees to break situational norms. Thus, the first purpose of this research is to examine the indirect effect of abusive supervision on unethical behavior via turnover intentions. 

Moreover, another insight of psychological contract theory highly related to our model is that individuals’ response to weak psychological contract in different ways depending on organizational context, such organizational context can modify the impact of turnover intentions on unethical behavior. One important factor that may impact coping style is organizational caring climate, unethical behavior researchers have found that “everyone for himself” climates are more likely to increase unethical behavior. In comparison, the reverse relationship is found where there is a climate that focuses on everyone’s interests [21]. Following these, the second purpose of this study is to investigate the moderating role of caring climate on the relationship between turnover intentions and unethical behavior. 

**Figure 1:**
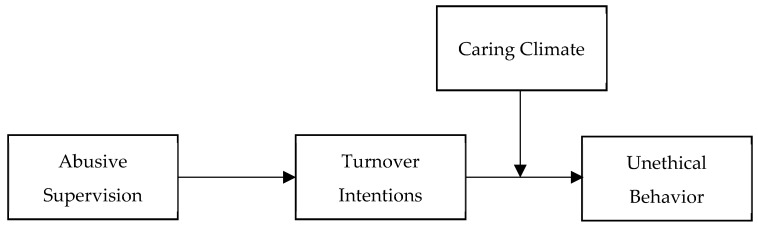
Theoretical model tested in this study.

In sum, the current research tests a theoretical model of the relationship between abusive supervision and unethical behavior and is designed to make three unique contributions. First, we draw from psychological contract theory to make a novel theoretical contribution that explains why relational aspects of supervisor–subordinate interactions affect subordinates’ behavioral responses to abusive supervision. An examination of this effect is likely to advance current understanding of the consequences of the dark side of leadership in organizations, and it also extends the limited understanding of the relationship between abusive supervision and employees’ unethical behavior. Second, by adopting a perspective based on psychological contract theory, we explore how turnover intentions mediate the relationship between abusive supervision and unethical behavior. This investigation advances the research by identifying a new underlying mechanism that explains the relationship between abusive supervision and unethical behavior. Finally, by illustrating the moderating role of caring climate, we recognize the boundary conditions which abusive supervision induces unethical behavior. This examination contributes to both the leadership literatures and the burgeoning studies on unethical behavior by demonstrating the ways in which organizational context can affect leadership processes and consequences. Figure 1 outlines the theoretical model tested in this study.

## 2. Theoretical Background and Hypotheses

### 2.1. The Mediating Role of Turnover Intentions

Abusive supervision refers to a destructive leadership style where employees perceive treatment from their supervisor as hostile verbal and/or non-verbal expressions [12], such as ridiculing, yelling at, and intimidating subordinates; taking credit for subordinates’ achievements; and attributing undesirable outcomes to subordinates’ personal factors [22,23]. Based on previous studies, we argue that psychological contract theory provides a new perspective in explaining subordinates who display unethical behavior in abusive organizational situations. 

Psychological contract theory is deeply influenced by the social exchange theory [24]. Further, it has been proposed that reciprocity is central to explaining the relationships between the evaluation of the psychological contract and employee attitudes and behaviors [24]. According to psychological contract theory [16], employees hold a specific set of beliefs regarding their relationship with the organization, which includes the reciprocal obligations of each party. As noted by Robinson [17], “psychological contracts refer to employees’ perceptions of what they owe to their employers and what their employers owe to them”. Researchers have shown that psychological contracts include both transactional and relational components [25,26]. The transactional component involves the more static aspects of the exchange relationship, such as pay and benefits. The relational component involves the more flexible aspects of the exchange relationship that presume a long-term relationship with the organization, such as training and development opportunities, promotion opportunities, and support from supervisors [27]. 

Employee turnover is defined as the cessation of membership in an organization by an individual who has received monetary compensation from the organization [28]. The turnover research has paid attention to the intentions and reasons behind turnover and to its consequences. For example, Mobley [29] provided a comprehensive explanation of the psychological process underlying turnover. In the process of making a withdrawal decision, individuals first evaluate their current jobs and experience satisfaction or dissatisfaction based on these jobs. A turnover intention can be formed if psychological attachment is decreased. 

Previous studies have clearly established that leadership can play an important role in construction and maintenance of employees’ psychological contract [30]. For example, when supervisors show abusive supervision behaviors to subordinates, subordinates will regard it as disrespect, contempt and humiliation to themselves. This is contrary to the expectation or belief that one should be treated fairly. It violates the exchange principle and ethics of “equality and reciprocity” between the two parties in the relationship, damages the positive image of the supervisors in the eyes of subordinates. That is, abusive supervision creates a low-quality relationship between supervisors and subordinates, which decreases the subordinates’ attachment and loyalty to the supervisor and organization. In this case, subordinates who do not feel that their supervisors care about them are unlikely to feel obliged to remain within the organization [13]. Moreover, subordinates are concerned about whether supervisors can provide them with sufficient learning and development opportunities and a just climate [31]. As abusive supervisors struggle to meet subordinates’ expectations, the relational contract between subordinates and supervisors or organization will be difficult to maintain. Similarly, a number of empirical studies have confirmed the positive relationship between abusive supervision and the turnover intentions of subordinates [32] Therefore, we hypothesize

**H1:**  *Abusive supervision will be positively related to subordinates’ turnover intentions.*

Turnover intentions signal that the relational contract between subordinates and the organization has been breached [33]. It stands to reason that, if an individual feels that this relationship has been breached, then they will develop strong intentions to quit. A subordinate with strong turnover intentions has effectively quit before officially leaving the organization and is emotionally disconnected from other employees and the organization, but such individuals should be less likely to be concerned about what their employer owes them or what they owe their employer in a relational sense [33]. Not only will such subordinates be less likely to help others, we argue that they will be more prone to behaving in ways that are counter to organizational policies, practices, and norms. Recent research reveals that turnover intentions result in increased incidents of negative work behavior [34]. We accordingly propose that turnover intentions impact subsequent unethical behavior and ultimately serve as a critical mediating mechanism explaining the relationship between abusive supervision and unethical behavior. Therefore, we hypothesize

**H2:**  *Turnover intentions is positively related to subordinates’ unethical behavior.*

We hypothesize a positive association between abusive supervision and subordinates’ turnover intentions. We also hypothesize that there is a positive relationship between turnover intentions and subordinates’ unethical behavior. Together, these hypotheses suggest that turnover intentions mediate the relationships between abusive supervision and subordinates’ unethical behavior. These mediated relationships are in line with research on psychological contract theory which has support the relationship between leadership and subordinates’ behavior. Therefore, we hypothesize

**H3:**  *Turnover intentions mediate the positive relationship between abusive supervision and unethical behavior.*

### 2.2. The Moderating Role of Caring Climate

There is reason to believe that the mechanism proposed in the above hypotheses may vary in its strength. Psychological contract theory [16] argued that organizational characteristics (e.g., organizational climate) affect how individuals cope with decreased psychological contract. Here, we propose that organizational climate will play critical roles in shaping the violation-behavior relationship. 

In organizations, employees work within a system of rules, regulations, procedures, and hierarchy [35]. The moral content of the organizational climate can have an impact on the moral development of the individual, and appropriate behavior can be learned by employees within their organization through climate perceptions. Rusaw [36] posited organizational climate is one of the organizational characteristics that influence individuals’ ethical decision. Researches also illustrate that the organizational climate serves as a behavioral norm for ethical judgments [37] and managerial moral decisions [38]. Ethical climate, which is one specific variable in the dimensions of climate, is a type of work climate that guides ethical behavior within an organization [39]. Ethical climate helps employees decide what is right and wrong. Victor and Cullen’s [40,41] initial work identified different types of ethical climate: professionalism, caring, rules, instrumental, efficiency, and independence. Fu and Deshpande [39] found that among these ethical climates, the caring climate had the strongest correlation with ethical behavior. Thus, we focus on this climate type in our study. Previous studies have also found that the caring climate has a significantly positive impact on various organizational outcomes, such as job satisfaction [42,43], supervisor satisfaction [44], job performance [39], and organizational commitment [45]. It is important to determine how organizations can deal with abusive supervision and the consequent unethical behaviors of employees. Therefore, this study tests the moderating effects of the caring climate. 

We propose that a caring climate can mitigate the positive effects of turnover intentions on unethical behavior. Within a caring climate, employees’ major consideration is the impact of utilitarian decisions on others rather than their self-interest [46]. Employees in a caring climate are also more likely to make decisions that are beneficial for the majority of people involved in the decisions [41]. Thus, we believe that a caring climate can offer cues for the expression of unethical behaviors and motivate employees to behave well. There has been indirect empirical support for our arguments. Research has found that employees’ negative affect may lead to deviant behavior, an effect that was significantly weakened when the caring climate was high [47]. Hence, for employees within a higher perceived caring climate, turnover intentions should have a constrained positive effect on unethical behavior, whereas employees within a lower perceived caring climate should be more likely to engage in unethical behavior. As a result, we suggest that the caring climate moderates the positive relationship between turnover intentions and unethical behavior. 

The prior arguments represent an integrated framework in which turnover intentions mediate the positive relationship between abusive supervision and unethical behavior and caring climate moderates the relationship between turnover intentions and unethical behavior. According to the notion that the caring climate moderates the relationship between turnover intentions and unethical behavior, and considering that abusive supervision is positively related to turnover intentions, it is logical to propose that the caring climate also moderates the mediating mechanism for turnover intentions in the relationship between abusive supervision and unethical behavior. As discussed above, a weaker relationship between turnover intentions and unethical behavior emerges in high-caring climate organizations, and the indirect effect of abusive supervision on unethical behavior through turnover intentions may also be weaker in such organizations. Therefore, we hypothesize

**H4:**  *Caring climate moderates the indirect effect between abusive supervision and unethical behaviors through turnover intentions. Specifically, the indirect effect is stronger when the organizational caring climate is low versus high, and the moderation effect occurs between turnover intentions and unethical behaviors.*

## 3. Methods

### 3.1. Procedure and Sample

The participants in this study were subordinates who worked at three high-tech enterprises based in Shanghai, China. The participants worked in a number of job domains and business functions related to the sales, product development, and after-sale services. Incomplete or inconsistent participant responses were seen as invalid or missing data, and thus were excluded in the survey. The final sample consists of 679 participants (estimated response rate of 79.9%); 58.8% of the respondents were male. Most of the respondents were aged 20–30 (73.6%) and 31–60 (26.4%), which gave us an average respondent age of 28.9 years old (SD = 5.1). Over half of the respondents had an undergraduate degree (67.5%). The participants had an average job tenure of 5.5 years (SD = 3.7) and an average organizational tenure of 3.8 years (SD = 2.6).

### 3.2. Measures

Abusive Supervision. Abusive supervision was assessed with Tepper’s [12] 15-item scale. The items were rated on a five-point scale ranging from 1 (extremely uncharacteristic of me) to 5 (extremely characteristic of me). Sample items ask whether one’s supervisor “… is rude to me,” “… makes negative comments about me to others,” “… reminds me of my past mistakes and failures,” and “… tells me I’m incompetent.” The coefficient alpha was 0.94 in the current study.

Turnover Intentions. Employees’ turnover intention was assessed with a five-item scale from Bozeman and Perrewe [48]. These five items were rated on a five-point Likert scale (5 = mostly true; 1 = mostly false). Sample items include “I will probably look for a new job in the near future,” “At the present time, I am actively searching for another job in a different organization,” and “It is unlikely that I will actively look for a different organization to work for in the next year (reverse scored).” The coefficient alpha was 0.89 in the current study.

Unethical Behavior. Unethical behavior was assessed with the 18-item scale developed and validated by Chen and Tang [49]. Each item was rated on a five-point frequency scale from 1 (never) to 5 (always). Sample items include “waste company time surfing on the Internet, playing computer games, and socializing,” “take no action against the fraudulent charges made by one’s company,” “Abuse the company expense accounts and falsify accounting records,” and “Overcharge customers to increase sales and to earn a higher bonus.” The coefficient alpha for the unethical behavior scale was 0.95 in the current study.

Caring Climate. The caring climate was assessed with the five-item scale developed by Victor and Cullen [40]. A five-point Likert scale (with 5 representing “strongly agree” and 1 representing “strongly disagree”) was used to measure the caring climate. Sample items include “The most important concern is the good of all of the people in the company as a whole,” “Our major concern is always what is best for the other person,” and “What is best for everyone in the company is the major consideration here.” The coefficient alpha was 0.89 in the current study.

### 3.3. Analytical Strategy

Prior to hypotheses testing, we used Mplus 7 to conducted a series of confirmatory factor analyses and examined multiple fit indices including Comparative Fit Index (CFI), Tucker–Lewis Index (TLI), Root Mean Square Error of Approximation (RMSEA), and Standardized Root Mean Square Residual (SRMR). Then, we used SPSS 21.0 to examine means, standard deviations, and inter- correlations for the study variables. Finally, we tested the hypothesized moderated mediation model using hierarchical regression analyses via PROCESS 2.16 for SPSS (IBM, New York, NY, USA) [50], bootstrapping was set to 5000 resamples. Additionally, because unstandardized coefficients are the preferred metric in causal modeling [50,51], all of the path coefficients are reported as unstandardized OLS regression coefficients.

## 4. Results

### 4.1. Confirmatory Factor Analysis (CFA)

Before testing our hypotheses, we first used Mplus 7 (Informer Technologies, New York, NY, USA,) to conduct a CFA and assess the distinctiveness of our key variables (abusive supervision, turnover intentions, unethical behavior, and caring climate). As shown in Table 1, the CFA results suggested that our four-factor baseline model fit the data substantially better than any of the alternative models, confirming discriminant validity (*χ^2^* = 2220.40, *df* = 854, CFI = 0.92, TLI = 0.91, RMSEA = 0.05, SRMR = 0.05). We next applied Fornell and Larcker’s [52] method check for convergent and discriminant validity. Table 2 shows the square roots of the average variance extracted for each latent construct. All the estimates exceeded the correlation between the factors comprising each pair. In addition, all parcels significantly loaded on the intended latent constructs and had standardized loadings above 0.5. Therefore, all the proposed constructs have good convergent and discriminant validity.

### 4.2. Common Method Bias

To check the problem of common method bias, Harman’s single-factor test [53] was conducted. The analysis returned seven factors with eigenvalues greater than 1, with the first factor explaining less than 50% of the variance (37.27% of 71.09%). In addition, a follow-up factor analysis with 4fourseparate factors showed that all of the factor loadings exceeded 0.50, which indicated a good inter-item reliability. In addition, we conducted a CFA by controlling for the effects of a single unmeasured latent method factor [53], the results showed that the five-factor model does not have a better data fitting than four-factor model. Thus, the findings provided no indications of common method variance.

### 4.3. Descriptive Statistics and Correlations

The means, standard deviations, correlations, and coefficient alphas for all of the study variables are presented in Table 2. Abusive supervision was positively related to turnover intentions (*r* = 0.40, *p* < 0.01), and turnover intentions were positively related to unethical behavior (*r* = 0.33, *p* < 0.001). Abusive supervision also exhibited a significant relationship to unethical behavior (*r* = 0.52, *p* < 0.001).

### 4.4. Hypothesis Testing

Hypothesis 1 predicted the relationship between abusive supervision and turnover intentions, Hypothesis 2 predicted the relationship between turnover intentions and unethical behavior, and Hypothesis 3 predicted an indirect effect between abusive supervision and unethical behavior through turnover intentions. These hypotheses were supported with significant direct effects and an indirect effect (see Table 3). There was a significant abusive supervision to turnover intentions direct effect (*a effect* = 0.39, *p* < 0.001), a turnover intentions to unethical behavior direct effect (*b effect* = 0.14, *p* < 0.001), and an indirect effect through turnover intentions (*indirect effect* = 0.055, CI_.95_ = 0.013, 0.098). An examination of the remaining direct effect of abusive supervision on unethical behavior (*c’ effect* = 0.47, *p* < 0.001) indicated partial mediation, supporting Hypothesis 1, Hypothesis 2, and Hypothesis 3.

**Figure 2:**
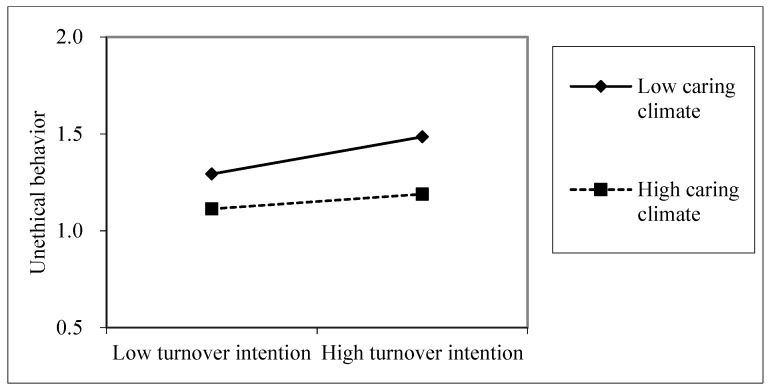
Interaction between turnover intentions and caring climate with regard to unethical behavior.

To test Hypothesis 4, which proposes that the indirect effect of abusive supervision on unethical behaviors through turnover intentions is conditioned by the level of caring climate, we conducted a moderated mediation analysis with caring climate as a moderator of the relationship between turnover intentions and unethical behavior. Support was found for this hypothesis with significant interaction terms in the moderated mediation models (see Table 3). There was a significant negative interaction term on the turnover intentions to unethical behavior path (*effect*= −0.03, *p* < 0.05). The interaction is graphically displayed in Figure 2. Additionally, the index of moderated mediation indicates that any two conditional indirect effects defined by different values of caring climate are statistically different (*index* = −0.011, CI_.95_ = −0.025, 0.001). 

To illustrate the presence of moderated mediation, we report the indirect effect at different levels of the moderator. Table 3 illustrates these moderated indirect effects through changes in the level of caring climate for unethical climate. Specifically, low levels of caring climate have a significant indirect effect (indirect effect = 0.022, CI_.95_ = 0.004, 0.040), while mean and high levels of caring climate have non-significant indirect effects (indirect effect = 0.010, CI_.95_ = −0.001, 0.030; indirect effect = −0.001, CI_.95_ = −0.030, 0.025). This means that abusive supervision has the strongest association with unethical behavior through turnover intentions when the level of the caring climate is low. 

## 5. Discussion

An understanding of ethical decision making in organizations is important for the development of organization science. It is thus meaningful to capture the important interfaces among individual and situational variables that together shape this complex phenomenon. However, research that uses an interactionist model of ethical decision making is limited. To address the above concerns, and in response to previous researchers’ [2,21] call for a person-situation interactionist model for understanding behavioral ethics, we developed the current study by investigating the relationship between abusive supervision and employees’ unethical behavior. Furthermore, we focused on two potential underlying mechanisms, predicting that employees’ turnover intentions would mediate this relationship and the caring climate to moderate the relationship between turnover intentions and unethical behavior. Overall, we found support for our hypothesized model. 

### 5.1. Theoretical Implications

Our research contributes to streams of literature on behavioral ethics and ethical decision making in several ways. First, by clarifying the relationship between abusive supervision and employees’ unethical behavior, we add a new perspective on unethical behavior and shed new light on the relationship between abusive supervision and unethical behavior. Reviewing the literature, we found that the previous research focuses on the inhibition of positive leadership on employees’ unethical behaviors [54]. However, few empirical studies have explored the direct influence of dark side of leadership on employees’ unethical behavior, thus leading us to pay attention to the effect of “praising virtue” but ignore the effect of “punishing vice.” Therefore, by drawing extensively from psychological contract theory, we focused on abusive supervision as an essential antecedent of employees’ unethical behavior. Previous research demonstrates that abusive supervision positively affects employees’ negative outcomes, such as counterproductive behaviors, supervisor-directed deviance, work-family conflict, psychological distress, and somatic health complaints [12,55,56,57,58,59,60]. However, research from an ethical perspective remains incomplete. We found evidence for a positive relationship between abusive supervision and employees’ unethical behavior. This result provides a wider perspective on the impact mechanism of abusive supervision and also provides support for the idea that the quality of the interpersonal treatment that employees receive from leaders is an important way in which leaders can influence followers’ unethical behavior [61]. Specifically, abusive supervision represents low- quality leader- employee relationships, it is contrary to the expectation or belief that one should be treated fairly, and it also violates the norms of reciprocity. In order to maintain the balance of relationship between the individuals and organization, employees may engage in covert and broader directed retaliation [62], such as unethical behavior, including behaviors such as falsifying expense reports, abusing sick time, and misusing company resources.

Second, our findings contribute to psychological contract theory by demonstrating that dark side of leadership provide a critical context that can induce subordinates’ unethical behavior via weakening subordinates’ psychological attachment towards organization. Previous studies focus on the psychological process of abused subordinates, for example, drawing on social cognitive theory, scholars indicated how social referents (i.e., abusive supervision) may influence subordinates’ unethical behavior through distinct process of moral agency [14]. It emphasized the important role of internalized self-standards and capacities for self-regulation on the mechanism between abusive supervision and unethical behavior. In addition, based on trait activation theory, the study indicated that abusive supervision can serve as a situational factor that activates the role of personality in predicting unethical behavior [8]. Unlike the prior researches, we used psychological contract theory, which emphasizes the reciprocal relationship between leaders or organizations and subordinates to illustrate that turnover intentions may be a crucial underlying mechanism that explain the positive association between abusive supervision and unethical behavior. Thus, we improve our understanding of which subordinates are likely to engage in unethical behavior in order to meaningfully balance relational processes between leaders and subordinates. 

Finally, by focusing on organizational characteristics—caring climate—as a moderator, this study builds a better understanding of the conditions under which unethical behavior is likely to occur. Unethical behavior in organizations is viewed as a consequence of both organizational and individual influences. However, empirical studies investigating the causes of unethical behavior are relatively few and have little to say about the interaction between the individual and organizational perspectives [3]. To fill this void, this research studies the moderating effect of the caring climate. As predicted, the results show that the caring climate plays a role in overriding the indirect influence of abusive supervision on unethical behavior. Specifically, the indirect effect between abusive supervision and unethical behavior was stronger for those in a lower caring climate. This result clarifies the findings of Hershcovis et al. [63] and suggests that the overarching role of the organization may not be sufficient to override the more immediate influence of the supervisor. 

### 5.2. Practical Implications

In practical terms, unethical behavior is costly for both managers and organizations in the Chinese context [64]. Our findings suggest two paths by which managers and organizations can minimize employees’ unethical behavior. The first is to take measures to reduce abusive supervision. Organizations should create a zero-tolerance culture regarding abusive behavior and offer abuse-prevention training for supervisors [65]. Moreover, research has revealed that relationship conflict leads supervisors to show abusive supervision [23]. Thus, organizations should encourage supervisors and subordinates to use suitable behavioral strategies to cope with relationship conflict. The second path to diminishing unethical behavior is to create a caring climate. A caring climate in an organization may prevent abusive supervision from resulting in unethical behavior on the part of employees. To increase the level of the caring climate, certain procedural designs and institutional arrangements should be set up in organizations, such as control over who is hired and promoted to a supervisory position. Employees who have displayed abusive traits toward coworkers need to be not only identified and stopped, but also prevented from rising to supervisory positions. 

### 5.3. Limitations and Future Directions

Although this study contributes to the literature in several ways, it also has a few limitations. The first design limitation is our use of self-reported measures, which may make our findings vulnerable to the effects of common method variance. We argue that given our research goal, a same-source data design was acceptable. First, a meta-analysis shows that self-reports of sensitive data (such as unethical behavior) are more accurate than data collected from other- reports [66]. Further, in view of employees’ deviant behaviors are concealed and difficult to be detected by others are concealed and difficult to be detected by others, self-reports are more accurate [67]. Second, scholars [68,69] have concluded that, if a moderating effect is found, it should be taken as strong evidence that CMV did not present a bias. Notably, our results show significant moderating effects and that the indirect effects varied over levels of the moderator variable. Though common method variance is not a serious problem in this study, this limitation should nonetheless be noted. Future research could examine these relationships with self and other reports to provide greater confidence in the study’s inferences. Second, we collected data at the same time in an effort to establish the temporal precedence of abusive supervision and unethical behavior. We also took steps to enhance the nature of causality in our study. We ordered the variables in the data collection efforts in such a way to ensure the causal chain was accurately represented. The ordering of variables in the model was based on strong theory, as well as experimental research. A future study could use a longitudinal design to confirm the casual relationship between abusive supervision and unethical behavior. Third, our data supported the importance of the caring climate in understanding the effect of abusive supervision on unethical behavior. However, it is also possible that the other boundary conditions may be used to explore the above relationship. Thus, we encourage future studies to contribute to the unethical behavior literature by examining the moderating impact of other variables. Finally, this study has provided insight into abusive supervision in the Chinese cultural context and responded to Wei and Si’s [70] call for further studies of the abusive supervision of Chinese employees. Future research may focus on national and cultural differences in abusive supervision [13], as cross-cultural research could establish whether large power distances and other cultural and institutional factors render Asian employees especially vulnerable to factors leading to abusive supervision [71].

## 6. Conclusions

Why do employees engage in unethical behavior when they are subjected to abusive supervision? As predicted, this research found that (1) abusive supervision has a significant direct impact on the unethical behavior of Chinese employees, (2) turnover intentions mediate the relationship between abusive supervision and unethical behavior, and (3) this indirect effect is conditional upon the caring climate. The testing of these mediation and moderated mediation mechanisms helped to answer the question above. Moreover, our findings extend the unethical behavior literature and suggest methods that managers can follow to reduce their employees’ unethical behavior.

## Figures and Tables

**Table 1:** Results of confirmatory factor analysis.

Model	χ^2^	df	CFI	TLI	RMSEA	SRMR
Four-factor model	2220.40	854	0.92	0.91	0.05	0.05
Three-factor model ^a^	2747.30	857	0.88	0.86	0.08	0.09
Two-factor model ^b^	3536.62	859	0.83	0.81	0.10	0.12
One-factor model ^c^	4139.71	903	0.78	0.76	0.13	0.14

^a^ This model combines turnover intentions and unethical behavior into one factor. ^b^ This model combines turnover intentions, caring climate, and unethical behavior into one factor. ^c^ This model combines all measurement items into one grand factor. Both three-factor model and two-factor model reported in Table 1 are optimal. CFI: Comparative Fit Index. TLI: Tucker–Lewis Index. RMSEA: Root Mean Square Error of Approximation. SRMR: Standardized Root Mean Square Residual.

**Table 2:** Descriptive statistics, correlations, and reliabilities.

Variables	*M*	*SD*	1	2	3	4	Square Root AVE
1. Abusive supervision	1.36	0.44	*0.94*				(0.81)
2. Turnover intentions	2.10	0.76	0.40 **	*0.89*			(0.78)
3. Caring climate	4.13	0.77	−0.43 **	−0.42 **	*0.89*		(0.75)
4. Unethical behavior	1.21	0.39	0.52 **	0.33 **	−0.39 **	*0.95*	(0.83)

*N* = 679. Cronbach’s alpha are in italics and appear on the diagonal; * *p* < 0.05 (two-tailed test); ** *p* < 0.01 (two-tailed test). AVE: Average Variance Extracted.

**Table 3:** Mediation and moderated mediation estimates.

Mediation Model						
Direct Effects	Coefficient	SE	*t*	*p*		Model *R^2^*
Turnover intentions as DV						
Constant	0.91	0.23	4.00	0.000		0.18 ***
Abusive supervision	0.39	0.04	11.11	0.000		
Unethical behavior as DV						
Constant	0.20	0.22	0.93	0.351		0.29 ***
Abusive supervision	0.47	0.04	13.04	0.000		
Turnover intentions	0.14	0.04	3.92	0.000		
Indirect effect	Effect	Boot SE	Boot LLCI		Boot ULCI	
Abusive supervision on unethical behavior	0.055	0.022	0.013		0.098	
**Moderated Mediation**						
**Direct Effects**	**Coefficient**	**SE**	***t***	***p***		**Model *R^2^***
Turnover intentions as DV						
Constant	0.91	0.23	4.00	0.000		0.18 ***
Abusive supervision	0.39	0.04	11.11	0.000		
Unethical behavior as DV						
Constant	1.31	0.08	15.74	0.000		0.32 ***
Abusive supervision	0.16	0.01	11.06	0.000		
Turnover intentions	0.03	0.01	1.80	0.072		
Caring climate	−0.07	0.01	−4.64	0.000		
Turnover intentions * Caring climate	−0.03	0.01	−2.55	0.011		
Conditional indirect effects	Effect	Boot SE	Boot LLCI		Boot ULCI	
Low caring climate	0.022	0.009	0.004		0.040	
Mean caring climate	0.010	0.010	−0.001		0.030	
High caring climate	−0.001	0.014	−0.030		0.025	
Index of moderated mediation	Index	Boot SE	Boot LLCI		Boot ULCI	
Turnover intentions * Caring climate	−0.011	0.006	−0.025		−0.001	

*N* = 679. Effect size estimates are unstandardized coefficients. Boot 5000 bootstrap samples, LLCI bias corrected lower limit confidence interval ULCI bias corrected upper limit confidence interval. *** *p* < 0.001. Turnover intentions * Caring climate is Turnover intentions × Caring climate, when testing the moderating role of caring climate, it means the interaction between Turnover intentions and Caring climate.
